# Supplementary material for: Conversion of mammalian cell culture media waste to microbial fermentation feed efficiently supports production of recombinant protein by Escherichia coli
Source: PLoS One. 2022 May 4;17(5):e0266921. doi: 10.1371/journal.pone.0266921 (PMC9067682; doi:10.1371/journal.pone.0266921)
Supplement: S1 Raw images — PDF of uncropped gel image from Fig 2, panel B. (PDF) [file pone.0266921.s001.pdf]

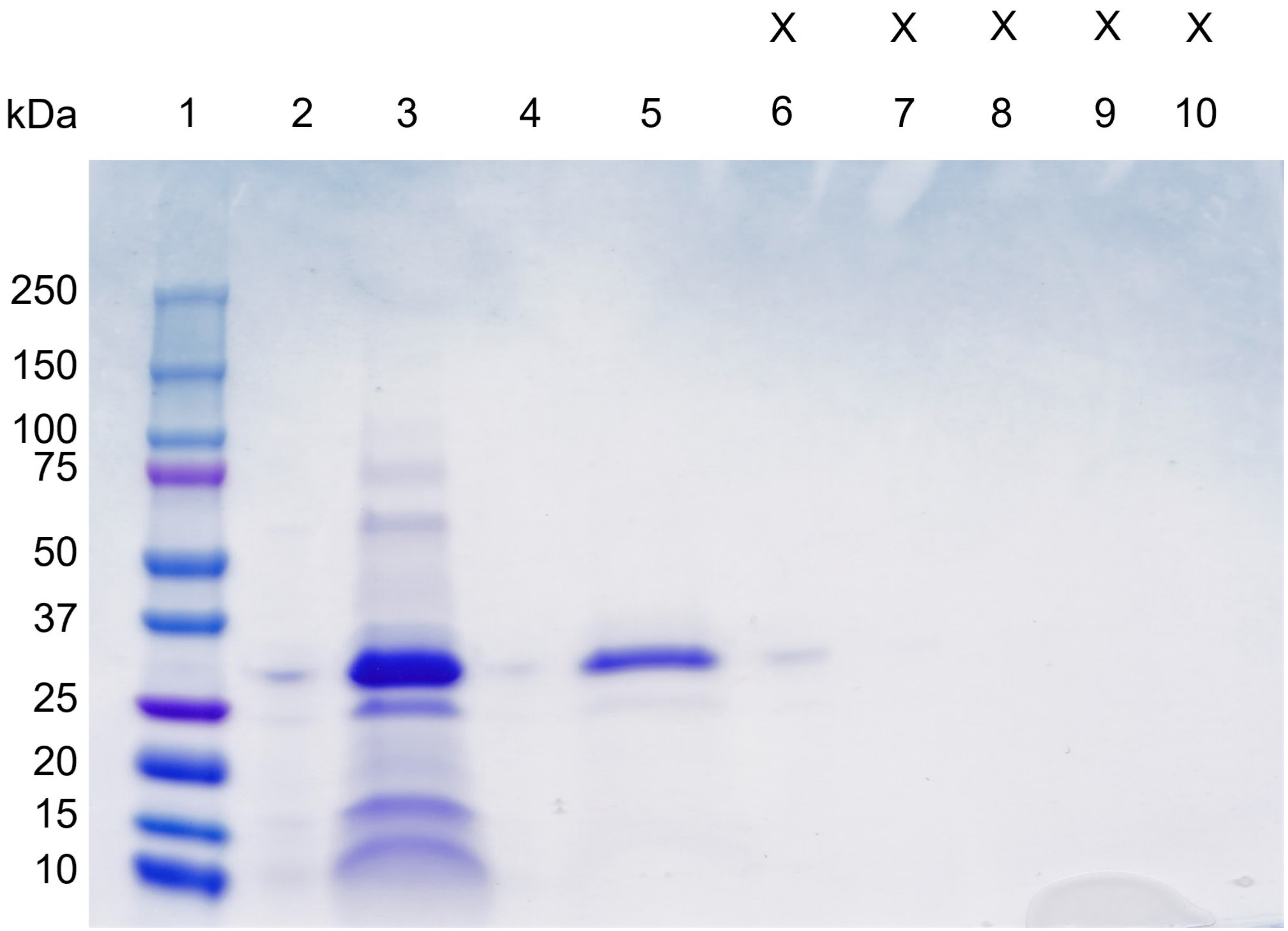

Lane 1; molecular ladder (Precision Plus Protein Dual Color Standards from BioRad), lane 2; blank, lane 3; post-boiled lysate fraction from protein purification, lane 4; blank, lane 5; size exclusion chromatography purified fraction, lane 6-10; blank. All lanes were loaded with 45 µl of sample mixed with 1X Laemmli Sample Buffer and denatured at 95 °C for 5 mins. Final figure in manuscript is Figure 2, panel B, lanes 1-5. Lanes marked with “X” not included in final figure. Image was captured by a CANON photo and document scanner.
